# Experiences of a Neurofeedback-Based Mindfulness Meditation Intervention for Migraine: Qualitative Study

**DOI:** 10.2196/68369

**Published:** 2025-07-31

**Authors:** Tora Levinton, Jan Gelech, Faly Golshan, Marla Mickleborough

**Affiliations:** 1 Department of Psychology and Health Studies, University of Saskatchewan, 105 Administration Place, Saskatoon, SK, S7N 5A2, Canada, 1 306 203 4155

**Keywords:** migraine, mindfulness, meditation, neurofeedback, behavioral intervention, mobile health, qualitative, headache

## Abstract

**Background:**

Migraine is a debilitating neurological condition often impacting the quality of life and resulting in physical, emotional, and social burdens. Pharmaceutical interventions are the conventional treatment for migraine; however, behavioral interventions provide safe alternatives. Both mindfulness meditation and neurofeedback are behavioral interventions that have been separately studied for migraine treatment. To date, no studies have investigated neurofeedback-assisted mindfulness meditation for migraine treatment and prevention.

**Objective:**

The objective of our study was to document the experiences of individuals with migraines who participated in an 8-week neurofeedback-based mindfulness meditation intervention as part of a randomized controlled trial.

**Methods:**

Semistructured interviews were undertaken with 10 participants (7 female and 3 male participants) aged 23 to 55 years who had previously completed an 8-week neurofeedback-based mindfulness meditation program using Muse wearable sensory headbands as part of a randomized control trial. The interview data were analyzed using reflexive thematic analysis.

**Results:**

Participants spoke to 3 categories of experiences: the positive impact of neurofeedback-based mindfulness meditation on migraine experiences, enhanced well-being and improved quality of life resulting from the intervention, and the benefits and drawbacks of incorporating a portable electroencephalogram technology into mindfulness meditation practices in the context of migraine treatment. In total, 9 participants felt that their ability to manage migraine symptoms was improved, and all participants expressed benefits beyond migraine prevention and pain management. Participants also spoke to the interconnectedness of migraine symptoms, daily stressors, and the framing of lived experience.

**Conclusions:**

Notably, as the first study to evaluate the experiences of individuals with migraines using an at-home, neurofeedback-based mindfulness meditation intervention, this investigation adds to our understanding of nonpharmaceutical migraine treatment. Participants reported that this neurofeedback-based mindfulness meditation intervention improved migraine management, leading to significant reductions in pain intensity, migraine frequency, and medication use. They also described improved quality of life and emotional regulation related to this intervention, which they attributed to enhanced attentional control and body awareness. This research supports the consideration of neurofeedback-based mindfulness meditation interventions using emerging technologies, such as wearable electroencephalogram devices, as an accessible behavioral intervention for migraine management.

## Introduction

### Background

Migraine, a debilitating neurological condition, manifests as recurrent attacks of moderate to severe pulsating head pain that is often localized to one side of the head and can last hours to days [1-3]. Symptoms include nausea, vomiting, and sensitivity to light and sound [2-4]. It is estimated that 14% of the world’s population and 8.3% of Canadian people experience migraine [3], negatively impacting quality of life and interfering with activities of daily living [2,5,6]. It is also frequently associated with emotional and social burdens, including mood disturbances, social isolation, anxiety, and challenges in work, family, and social contexts [5-8]. Migraine is one of the 2 leading causes of years lived with disability in high and middle sociodemographic index countries and one of the 5 leading causes of years lived with disability globally, with an average prevalence in 2016 of 1.04 billion [9].

Prescription and over-the-counter medications are the conventional abortive and preventive treatments for migraine [10]. Unfortunately, these medications come with negative side effects such as dizziness, drowsiness, nausea, and the risk of developing medication overuse headaches—classified as overuse of therapeutic medication leading to chronic headache symptoms in individuals prone to migraine [10-13]. Behavioral migraine treatments such as neurofeedback and mindfulness practices may offer safe and effective alternatives or complements to medication [14,15].

Mindfulness-based practices involve purposefully directing one’s attention to the present moment and the experience of various sensory, emotional, and cognitive events with an attitude of nonjudgment, curiosity, and acceptance [16-18]. Mindfulness-based interventions are popular and effective behavioral interventions for migraine [19]. Quantitative, mixed methods, and qualitative studies have reported significant decreases in migraine pain intensity [20,21], frequency [4], and medication use [22] associated with these interventions. In a recent unblinded trial, participants reported reduced monthly migraine days and acute medication use after a 10-day mindfulness meditation retreat [23]. A longitudinal study by Grazzi et al [24] argued that mindfulness-based treatments were comparable in effectiveness to prophylactic medication use among individuals with medication overuse headaches in terms of headache frequency and medication intake. The psychosocial benefits of mindfulness meditation are also evident among individuals with migraine, including reduced migraine-related disability [23-25], improved quality of life [26], and increased pain tolerance [27].

While effective, mindfulness meditation has a few limitations as a treatment for migraine. Mindfulness meditation is a time-intensive intervention; research suggests that an effective “dose” requires a daily practice of 20 minutes or more [10,28]. Individuals with migraine may also struggle to access trained educators and service providers or to locate evidence-based resources to develop effective mindfulness meditation skills [29]. The plethora of resources available to consumers, coupled with the intangible nature of mindfulness meditation practice and difficulties documenting skill progression (particularly among beginners), can also serve as barriers to successfully implementing mindfulness meditation to treat migraine pain [30]. Researchers also note that a lack of clarity around ideal dosage patterns, session durations, delivery methods, and responder characteristics continues to hamper this treatment approach [4].

Neurofeedback involves observing real-time displays of brain activity (typically using an electroencephalogram [EEG]) and learning how to alter one’s brain activity to achieve a calmer state [28]. Widely used in clinical practice for migraine [19], review papers have noted a relationship between EEG-based neurofeedback programs and improvements in migraine frequency, pain intensity, and indicators of disease burden, such as fatigue and anxiety [31,32]. Recent quasi-experimental studies have also reported significant reductions in pain [33], stress, and anxiety [34] as well as improved sleep quality in individuals with migraine [35] related to neurofeedback. Moreover, studies have suggested that clients using both neurofeedback and medication are more successful in reducing migraine frequency compared to those using medication alone [34].

However, existing research has been limited by high protocol heterogeneity [36], and review studies suggest that it is unclear which neurofeedback protocols would best suit particular patients with chronic pain [31]. Consequently, scholars recommend integrating neurofeedback with other interventions, like relaxation training or mindfulness meditation, mainly because the integration of such techniques is shown to regulate cortical activities, resulting in an improved headache experience [37,38]. Neurofeedback-assisted meditation regimes are seen as promising approaches to address neurofeedback delivery heterogeneity [39]. However, research in this area is limited. Although a meta-analysis by Darling et al [40] provided moderate support for the use of neurofeedback-assisted relaxation training in treating migraine conditions in pediatric patients, the included studies were limited, in that few studies were migraine-specific and were dated [40]. Neurofeedback-assisted meditation training for migraine in adults remains largely unexplored.

The use of neurofeedback for migraine treatment is also limited by various pragmatic challenges, including the availability of trained service providers and specialized equipment and notable time demands. Effective sessions take up to an hour at a time, and treatment protocols include numerous weekly visits over the course of several months [19,41]. Thus, it can be a costly and time-consuming treatment option. Further, high treatment heterogeneity and patient training protocols and objectives that are arguably even more intangible than those associated with mindfulness-based practices (focusing on the breath is slightly more concrete than modulating one’s brain activity) represent additional barriers to effectively implementing this therapeutic approach [30,31,36].

In short, mindfulness meditation and neurofeedback are both thought to benefit people with migraine and may have additive value when used together as an alternative or complement to medication. However, the success of these treatments depends on regular practice, access to trained instructors, and evidence-based information, and in the case of biofeedback, regular use of specialized equipment [15,42,43]. Financial costs, time constraints, and access issues represent significant barriers to the widespread use of behavioral migraine interventions. Despite widespread recommendations for combining their use and optimism over the efficacy of combined protocols, no studies to date have investigated the use of mindfulness meditation with neurofeedback in treating adults with migraine. Further, there is little consensus regarding best practices in using neurofeedback, mindfulness meditation, or a combined protocol to treat migraine or which patients might benefit most from these interventions. Although rapidly evolving portable technologies are making behavioral interventions more accessible to people with migraine, additional research on user expectations, experiences, and preferences, clinical outcomes, and best practices is needed to better appreciate whether these technologies are meeting the needs and health objectives of the diverse migraine experience.

### This Study

This study is part of a broader program of research on the impact of at-home neurofeedback-assisted mindfulness meditation on the migraine experience. At-home neurofeedback-assisted mindfulness meditation facilitates learning through guided sessions and feedback on brain states and encourages treatment adherence through records of session history and progress in attaining meditative states over time [44]. In doing so, this at-home intervention has the potential to address access issues and overcome some of the limitations of standalone consumer-grade neurofeedback or mindfulness meditation [19,30,31,36]. In this retrospective qualitative study, we interviewed individuals with migraine who had completed an 8-week neurofeedback-based mindfulness meditation intervention using a portable system for neurofeedback mindfulness training as part of a randomized controlled trial [45]. Qualitative investigations are increasingly being used to complement RCTs and provide a deeper understanding of intervention experiences, acceptability, meanings, and processes [46,47]. In line with this, the aim of this study was to ascertain how adults with migraine experienced an at-home neurofeedback-assisted mindfulness meditation practice, its perceived impact on migraine symptoms, and how it affected their daily lives and coping. We also explored how this intervention impacted participants’ quality of life and ability to complete activities of daily living. Participants were queried about the benefits and limitations of the intervention and whether they had continued engaging in mindfulness meditation with neurofeedback after the intervention period. In what follows, we delineate the process of making sense of the experiences of individuals with migraine with an at-home neurofeedback-based mindfulness intervention.

## Methods

### Ethical Considerations

This study was approved by the Human Ethics Research Board at the University of Saskatchewan (REB 1987). All participants provided informed written consent prior to data collection. Participants were informed of their right to withdraw from the study at any time throughout the trial. To protect participant privacy, all data were deidentified, stored on secure, password-protected servers, and accessible only to authorized research personnel. After data collection, participants received a debriefing summary outlining the study’s purpose and procedures. As compensation for their time and participation, individuals were allowed to keep the portable neurofeedback devices used during the study.

### Participants

Participants were recruited from a larger group of 64 individuals with migraine who had participated in a study of the effects of a neurofeedback-based mindfulness meditation intervention with Muse on migraine severity, migraine disability, headache management self-efficacy, and comorbid psychiatric disorders. This group consisted of adults with either a migraine diagnosis or headache symptoms that met the International Classification of Headache Disorders [1] criteria for migraine disorder who had smartphone and internet access. Of these, 33 completed a neurofeedback-based mindfulness meditation task, and 31 completed an attention control task. Exclusion criteria included having started a new preventive medication within the last 6 months [48], comorbidity of Raynaud syndrome or diabetes, and previous engagement in meditation practices (ie, regular meditation sessions exceeding 60 minutes per month).

Those in the meditation condition underwent a 56-day intervention that involved using the Muse system once a day. Daily sessions started with the syncing of the headband to the Muse app and the calibration of the headband technology. Next, participants undertook 1 of 10 guided introductory, instructional mindfulness meditation recordings available in the Muse app, each lasting 2‐3 minutes (consumed in order for the first 10 days and then based on participant preference thereafter). These recordings provided instructions for how to sit for meditation and how to monitor, accept, or influence bodily sensations, breath, emotions, or thoughts. Next, participants used the immersive soundscape function for a 10-minute unguided meditation session with neurofeedback. In this neurofeedback session, different auditory cues within the Muse app indicated active, neutral, or calm brain states. Based on this auditory feedback, participants used the specific mindfulness meditation skills they learned in the instructional recording to pursue more calm brain states (indicated by the auditory feedback). At the end of these sessions, participants were provided with a report of the percentage of time spent in different brain states (active, neutral, or calm) according to the EEG data. All activities were undertaken within the free version of the Muse app, and all participants reported having completed all daily sessions.

Additionally, participants completed detailed questionnaire-style migraine headache diaries on the web each time they experienced migraine symptoms throughout the intervention period. These diaries queried information related to the onset (such as the time they noticed indicators of an oncoming migraine and migraine expectation—if they could predict the onset of migraine based on symptoms or triggers), nature (such as peak intensity, average intensity, and disability for the day), duration of pain (such as the time attack started and finished), participants’ emotional state during these symptoms (such as stress, anxiety, irritability, happiness, sadness, anger, boredom, relaxation, and poor concentration), possible triggers (such as dietary, sleep, hormonal, environmental, physical, stress, and stress letdown), and any treatment techniques used to manage symptoms (such as resting in a quiet dark place, taking over-the-counter medicine, massaging the scalp, applying a cold compress, and drinking herbal tea).

For this study, participants who had completed the neurofeedback-based mindfulness meditation intervention with the Muse system were invited to undergo additional semistructured interviews regarding their experiences of this intervention. Interview data served as the basis of an interpretative phenomenological investigation [49] into how adults with migraine experienced an at-home neurofeedback-assisted mindfulness meditation practice. Potential participants were contacted by email, and no additional inclusion or exclusion criteria were applied. The first 10 individuals who agreed to participate were interviewed by the first author (TL). All current participants identified as White, and the majority were female participants (n=7). Ages ranged from 23 to 55 years, with a mean age of 32.6 (SD 9.59) years. With regard to employment status, 7 participants indicated that they were students, homemakers, or volunteers, 1 noted they were unemployed or retired, and 2 chose not to answer. Though small, this sample size is consistent with the goals and purposes of interpretative phenomenological analysis [49].

### Data Collection

Semistructured interviews were conducted with participants in December 2022. All interviews were conducted over Zoom (Zoom Video Communications), lasted between 20 and 70 minutes, and explored experiences of neurofeedback and mindfulness meditation with Muse. Participants were asked about the intervention’s effects on their migraine symptoms, migraine frequency, coping mechanisms, quality of life, and activities of daily living. Participants were also asked about the influence of Muse on their meditation experience, their perceptions of the strengths and limitations of the system, and their intentions for future use of the Muse system and meditation techniques learned during the study. Interviews were audio recorded and subsequently transcribed by the first author (TL). Information on migraine and headache frequency was collected from participants before the intervention, with migraine frequency (monthly) ranging from 0.00 to 9.33, with an average of 3.09 (SD 2.89), and headache frequency (monthly) ranging from 0.33 to 11.67, with an average of 4.42 (SD 5.22). The time (months) between the end of the official intervention period and the interview ranged from 8.00 to 13.57 (mean 9.4, SD 4.54).

### Data Analysis

Per the 6 steps of reflexive thematic analysis outlined by Braun and Clarke [50,51], the authors first familiarized themselves with the data. Data were then assessed for patterns (codes) of manifest and latent meanings using an inductive or bottom-up approach (though researchers’ interpretations of the data were invariably influenced and nourished by their familiarity with existing literature). Codes were then assessed for recurring patterns and developed into a preliminary map of codes and themes, with themes representing a higher level of abstraction and codes fitting within one or more themes. This map was then refined in an iterative process of returning to the data and the literature and generating clear and precise theme descriptions as well as more abstract categories and more specific subthemes. The final step involved integrating these experiences into a cohesive structure that conveyed the relationship between categories, themes, and subthemes and the essence of the data as a whole.

## Results

### Overview

In sharing their experiences of neurofeedback-based mindfulness meditation using the Muse system, participants broadly spoke to 3 categories: the positive impact of mindfulness meditation with Muse on migraine experiences, enhanced well-being and improved quality of life resulting from the intervention, and the benefits and drawbacks of incorporating Muse technology into mindfulness meditation practices in the context of migraine treatment. In what follows, we elaborate on participants’ experiences according to these 3 categories of information. Table 1 summarizes the key findings of the study, detailing categories, themes, and subthemes.

**Table 1.** A summary of key findings.

Category and theme		Subtheme	Values, n (%)
Positive impacts on migraine experience			
	Improved migraine management and prevention	Reduced frequency of migraine episodes	6 (60)
	Improved migraine management and prevention	Improved ability to prevent severe intensity migraine pain	9 (90)
	Improved migraine management and prevention	Better able to cope with acute migraine symptoms	9 (90)
	Transforming the experience of migraine	Reduced fear of migraine pain	5 (50)
	Transforming the experience of migraine	Feeling more empowered	7 (70)
	Transforming the experience of migraine	Developing a more constructive understanding of migraines	9 (90)
Enhanced well-being and improved quality of life			
	Improved psychoemotional well-being	—^a^	8 (80)
	Enhanced social relationships	—	7 (70)
	Establishment of healthier daily routines	—	6 (60)
	Enhanced appreciation for holistic health	—	9 (90)
	Fewer migraine-related disruptions to daily life	—	4 (40)
Perceived benefits and drawbacks of the portable EEG^b^ device			
	Benefits of meditation with a portable EEG device	Session-tracking promoted adherence	9 (90)
	Benefits of meditation with a portable EEG device	Live EEG data were helpful in developing a meditation practice and tracking personal progress	10 (100)
	Drawbacks of the portable EEG device	Technological and instrumental challenges	10 (100)
	Drawbacks of the portable EEG device	Live auditory neurofeedback alerts were distracting	8 (80)
	Drawbacks of the portable EEG device	Perceived incongruencies between meditation experiences and EEG records were frustrating	8 (80)
	Drawbacks of the portable EEG device	The headband was uncomfortable during active migraine symptoms	5 (50)

a Not available.

b EEG: electroencephalogram.

### The Positive Impact of Mindfulness Meditation With Muse on Migraine Experiences

In total, 9 of 10 participants reported significant improvements in their migraine experiences related to the neurofeedback-based mindfulness meditation intervention. These improvements included enhanced migraine management and prevention capacities as well as positive shifts in the experience of migraine.

#### Improved Migraine Management and Prevention

In total, 9 participants reported that neurofeedback-based mindfulness meditation with Muse improved migraine management and prevention by reducing the frequency of migraine episodes (6/10), enhancing their ability to prevent severe-intensity migraine pain (9/10), and providing effective strategies for coping with acute migraine pain (9/10). Speaking broadly to decreases in episode frequency and intensity, Participant 4 noted: “since I’ve done that practice [mindfulness meditation], I’ve had less frequent migraines ... [and] my migraines—they’re not as bad as they used to be.”

Participants largely attributed reduced migraine frequency and intensity to reductions in psychological stress and accompanying bodily tension through mindfulness meditation with Muse (9/10). Speaking of the relationship between stress and migraine attacks, Participant 9 explained that learning to calm the mind and relax the body on an ongoing basis served to reduce migraine frequency: “[The intervention] helped me, like, deal with outside stress ... like [I experienced] less stress in general.” Many (7/10) also noted that the increased interoception (awareness of internal body signals) they developed as a result of this practice increased their awareness of early signs of an impending attack and improved their ability to identify possible triggers (including poor posture, certain repetitive motions, muscle tension in certain areas, prolonged immobility, stressful experiences, inadequate sleep, and dietary behaviors). In the words of Participant 3:

You can go by without even realizing where your pain is or what’s going on in your body. So that was an opportunity for me to do that at least once a day, and just get that centering. And I think that helped me become more aware of my body in general, of some of the triggers that brought out a migraine.

By avoiding such triggers or taking preventative action at the earliest signs of migraine, participants felt that they were able to effectively reduce the frequency of “full-blown” migraine attacks (characterized by moderate to severe intensity pain; 7/10). For example, Participant 8 described how becoming attuned to early warning signs of an impending migraine and prophylactically redirecting their attention while engaging in mindful breathing allowed them to prevent several migraine episodes: “I could ... stop my migraine without painkillers with diverting my attention ... I was like, take a deep breath, think about something else, and it was gone, and it never started that day.” Participant 7 similarly explained how mindfulness meditation, in combination with the headache diary, allowed them to better identify and avoid migraine triggers, thus reducing the frequency of their occurrence: “I started to kind of notice the triggers more. Especially when I was doing those [migraine diaries] ... I would notice ‘okay, this could be stressful, or this could give me a headache’, and I would just stop.”

These same factors (global reductions in psychological stress or bodily tension, increased awareness of specific triggers, and becoming more attuned to bodily signs of impending migraines) were also considered central to participants’ sense of being better equipped to recognize early warning signs and initiate early behavioral responses that either prevented or reduced the intensity of the migraine attack. For example, by removing themselves from sources of agitation and initiating prophylactic pharmaceutical treatment, Participant 4 felt that they were able to prevent high-pain episodes and return to a state of wellness more quickly: “Before [the intervention] migraine would [mean I was] done the whole day ... I would just shut off from the world until it stopped. [Now], I take that break, and I focus on myself and ... then the pain reduces.” Participant 9 similarly noted experiencing fewer high-intensity migraines since developing these skills: “the *severity* has been less.”

In addition to reflecting on enhanced prevention efficacy, 9 participants also described using mindfulness meditation (with or without the Muse system) as a means of coping with acute migraine pain. Coping refers to voluntary thoughts and behaviors mobilized to manage internal and external stressful situations [4]. For example, Participant 7 described using mindfulness meditation with the Muse system outside of scheduled intervention sessions to cope with acute migraine pain: “it help[ed] with decreasing the pain ... when I meditated ... it sort of overtook that [migraine] pain.” Participants proposed various theories about how mindfulness meditation was able to diminish migraine pain. In total, 7 participants attributed this ameliorative effect to enhanced attentional control and the ability to divert one’s attention away from physical pain. For example, Participant 10 noted: “[For pain management], I would try to refocus just on my breath. So, I wasn’t thinking about anything except for the breath.” Participant 4 similarly described how the intervention taught them to divert their attention away from migraine pain to achieve relief: “I ... interrupt the pain or focus on something else [mantra and breath] and divert that pain ... [to] have some temporary relief.”

Additionally, 5 participants noted developing the ability to separate themselves from embodied experiences of pain, known as psychological decoupling (mechanism of disengaging the physical aspects of pain from the emotional aspects of pain, leading to a reduced experience of distress through nonattachment [4]). Psychological decoupling leads to the ability to refocus attention, allowing individuals to experience lower pain through replaced focus, as Participant 1 noted: “letting go of ... [tensions] that are causing you pain ... removing yourself from, like, the experience of having pain ... a form of relaxing ... distancing myself from the stimulus of the pain.”

Finally, 8 participants felt that the pain-reducing capacity of mindfulness meditation was tied to its ability to promote a state of profound psychological and physical relaxation. Participant 4 noted that the practice helped to “relax the brain” and reduce muscle tension in ways that diminished migraine pain. Participant 1 similarly explained that mindfulness meditation was about “letting go” of the many tensions “that are causing you pain.”

As the participants developed and refined migraine prevention and pain management techniques throughout the intervention period, 6 participants reported reducing their use of pharmaceutical painkillers. Participant 5 noted that since the intervention, their “first jump isn’t to the medication” anymore, further explaining that “I didn’t take any medication because it didn’t even occur to me to take it ... [during a migraine] I laid back down and ... I did my breathing [mindfulness meditation technique].” In addition, Participant 2 stated: “Once I got used to the meditation and kind of knew how I like to do it, and what kind of environment I like to be in, I would kind of reach for almost that instead of going to a medication.”

Emboldened by alternative coping and pain management strategies, 3 participants spoke about delaying medication intake to see if they could manage the episode without pharmaceuticals. Participant 7 described: “if it’s not as severe or I didn’t think it would be as severe, I would take less. Or I would try first [to manage the pain] without it.”

Overall, the participants perceived the intervention as helpful for reducing migraine frequency and pain intensity through enhanced stress management, interoception, awareness of triggers, and earlier intervention. They also noted that neurofeedback-based mindfulness meditation benefited acute coping through enhanced attentional control and psychological decoupling. Thus, most participants felt that the intervention had improved their migraine symptoms and coping efficacy. As Participant 3 noted, learning new prevention and pain control strategies “helped me be able to cope with it [migraine], be able to understand it, and navigate it a little bit better than I used to.”

#### Transforming the Experience of Migraine

In total, 9 participants described qualitative changes to their experiences and understandings of migraine that they associated with mindfulness meditation with Muse. As they learned to better predict, prevent, and cope with migraines throughout the intervention, these individuals described a reduced fear of pain (5/10), an increased sense of personal empowerment in living with migraines (7/10), and developing more constructive understandings of migraines (9/10). Living with an unpredictable, treatment-resistant, and debilitating chronic illness can be very stressful. As Estave et al [9] explained, part of the distress implicit in living with migraine and headache conditions is the anticipatory anxiety (AA) that comes with wondering when a migraine will occur, how painful and debilitating it might be, and whether it will respond to interventions.

Although none of the participants described meditation with Muse as having prevented all migraine attacks or fully eliminated migraine pain, many noted that developing even limited efficacy in preventing and treating migraines using mindfulness meditation reduced AA and increased feelings of self-efficacy in managing these conditions. In total, 5 participants described worrying less about the possibility of developing a migraine. For example, Participant 4 described how being better able to manage migraine pain through meditation practices helped dissipate a lot of the anxiety around possible episodes: “That dread went away ... I knew that I could try something different to divert it ... like ‘oh, I’m going to get it [migraine pain] but it’s not going to be that bad or I can do different things to mitigate it.’”

Participant 2 similarly described how feeling more capable of managing this condition reduced the sense of living with a wholly unpredictable and uncontrollable body: “[It is a] mind over body kind of thing, like I have the power to control my brain and so now that I am more attuned to it [the state of mind and body], I feel like I could kind of control how I was feeling ... I now had something [mindfulness meditation] that would help me feel better, that I knew helped me feel better.”

Though all participants acknowledged that migraine symptoms would be an ongoing part of life, 7 participants noted that the sense of being able to effectively engage in some level of pain control and management reduced the fear around impending episodes. This sense of having even a modest degree of influence over the body and its symptoms not only reduced AA but also transformed individuals with migraine from passive survivors into active agents of their own well-being. Speaking about this developing sense of personal agency, Participant 7 stated, “it felt empowering—that I was somehow in control [of my migraines].”

In total, 9 participants described how becoming more attuned to embodied distress and environmental migraine triggers altered their perspective on migraine pain. As they grew more aware of the connections between migraine pain and a host of environmental, relational, and lifestyle stressors, these individuals came to understand migraine pain as an embodied sign that life was not in balance. Participant 3 noted, “[I started] seeing [migraine pain] less as an intrusion in my life, and more so as part of my life, or at least an expression of something going on in my life.” In doing so, these individuals recast the body and its pain as informants and allies, capable of providing important insight into personal well-being and warning the individual about the need to slow down or re-establish balance. Continuing, Participant 3 explained: “I didn’t feel that the meditations directly *reduced* my pain, but it affected how I *related* to that pain.”

### Enhanced Well-Being and Improved Quality of Life From the Intervention

In speaking about how mindfulness meditation with the Muse system influenced their daily lives, all participants described benefits beyond migraine prevention and pain management. Participants reported that mindfulness meditation with Muse benefited their psychological well-being, interpersonal relationships, work performance, and daily habits. In total, 8 participants noted general improvements in their psychological and emotional well-being as a result of mindfulness meditation, including reduced stress, a decrease in intrusive thoughts, an enhanced ability to achieve physical and mental relaxation, global improvements in mood, and greater energy, focus, and motivation throughout the day.

In total, 7 participants also reported that engaging in neurofeedback-based mindfulness meditation using the Muse system benefited their relationships and support systems. Participant 7 reported that participating in the intervention increased the visibility of prevention and coping efforts and occasioned more family conversations about migraine: “the kids became more aware of that [living with migraine] ... [that I] sometimes ... get sick.” Participant 2 noted that the practice aided in emotional regulation, leading to fewer relationship difficulties: “I think definitely on the days where I did meditate ... thoughts are clearer ... [I could] get my, like, point across in discussions ... if I forgot to meditate ... I did feel like I would get more confrontational.”

In total, 6 participants also felt that the intervention helped them establish healthier routines and sleep-wake cycles. For example, Participant 1 noted how getting up each day to complete the meditation exercise: “I would do the meditations every day ... first thing in the morning and so that ... helped build a better structure in my day that otherwise wouldn’t have been there.” Alternatively, participants who added mindfulness meditations to their evening routines felt that it helped them to relax and prepare for sleep in ways that benefited sleep hygiene and quality. Participant 2 noted that it “helped me go to sleep ... brings my brain down to like the normal speed ... [makes] the end of the day ... feel like the end of the day ... and then sleep would come, like, in a more natural sense.” Regardless of when participants meditated, the practice of taking 10 minutes each day to check in with the body, experience a quiet moment alone, and engage in a process of relaxation was deemed beneficial by all but 1 participant. Aside from any specific benefits to the migraine experience or one’s cognitive, emotional, or social well-being, the mindfulness meditation intervention encouraged participants to spend a few moments engaging in “self-care” each day. As Participant 7 noted, the mindfulness meditation “provided a bit more different routine ... something to look forward to ... sometimes that’s all you need to just kind of, like, relax.”

Often, discussions of the intervention’s benefits incorporated interrelated aspects of mind, body, and wellness. Participant 10, for instance, noted that the practice benefited his migraine symptoms, stress levels, and chronic muscle tension: “[Mindfulness meditation] would give me some relief overall, not just with migraines, but also with stress ... I have a lot of tension in my upper shoulders and neck area as well as my glutes, and ... everything relaxed more.” Importantly, these diverse benefits were often said to have an interactive, additive effect, given the interconnectedness of mind, body, environment, and circumstances. In discussing such interactions, 9 participants spoke to a “positive domino effect,” wherein proximal outcomes of checking in with and becoming more attuned to mind and body states lead to distal outcomes of positive lifestyle changes to improve mind and body states [4]. For example, 9 described how attending to the state of their minds and bodies during mindfulness meditation with live biofeedback led to sobering realizations about routine stress experiences and the inability to separate mind and body. Participant 9 expressed how they noticed “how stressed I was when I would approach the meditation ... I think, like, just being more cognizant of like how my body was feeling [during the meditation] ... in response to my mind was really huge.” Such realizations prompted numerous lifestyle changes intended to eliminate or reduce chronic stressors, including reducing media consumption, stepping back from stressful activities, and seeking out restful activities in the evenings. Participant 7 noted, “I think [mindfulness meditation] really helped with kind of controlling the environment that might actually contribute to [migraine].” Here, meditation draws awareness to stress states and spurs practical life changes that subsequently benefit migraine symptoms by reducing daily stress and exposure to triggers. In such cases, mindfulness meditation with the Muse system helped individuals with migraine to appreciate the interpenetration of mind, body, and experience and spurred the pursuit of a healthier lifestyle.

Participants also described how an increased ability to prevent and manage migraine pain reduced disruptions to everyday life, allowing them to perform better at work and fulfill commitments in their personal lives. For instance, 4 participants noted that increased awareness of migraine triggers enabled them to optimize their work schedules and avoid taxing experiences throughout the day, thereby reducing absences and increasing performance. Participant 7, for example, described undertaking more strenuous work tasks at the beginning of the week and reducing responsibilities as the week wore on to avoid triggering weekend migraines. Fewer disruptions to activities of daily living meant that participants not only felt more in control of daily life but also experienced less worry, frustration, and guilt and thus reduced their ongoing exposure to stress-related migraines. As Participant 5 noted, a reduction in the frequency of migraine episodes led to fewer missed days at work: “Normally I would probably have ... two to three pretty bad ones that I would actually have to miss work for ... and I’ve only missed one day of work for migraine [since beginning the intervention].”

In this sense, mindfulness meditation helped to disrupt a negative loop, wherein the activity limitations of chronic pain increased everyday stress, which subsequently increased migraine frequency. Speaking about how enhanced pain management allowed them to complete activities of daily living and avoid the psychoemotional strain of unfinished projects and unmet obligations, Participant 7 noted: “meditating helped with decreasing the headache, then, of course, automatically, I would be better at concentrating and fulfilling that [work and life tasks].” Participant 5 similarly noted how mindfulness meditation left them feeling more driven and optimistic, which, in turn, allowed them to access social supports and leisure activities that increased their quality of life: “my motivation levels have increased significantly ... [I have] the energy to ... get through some stuff [after-work activities] ... that is a visible change from before [the intervention].” Here, we see how enhanced pain prevention and management benefited daily quality of life in ways that reduced global stress and daily disruptions, reducing exposure to migraine triggers. In short, throughout the course of the intervention, pragmatic coping skills, personal insights, and lifestyle changes interacted in dynamic ways to enhance the quality of life of participants.

In addition, neurofeedback-based mindfulness meditation not only improved migraine experiences for the majority of participants but also benefited the quality of life for individuals with migraine. These dual benefits were not independent but rather interacted in complex ways to benefit participants. Where meditation helped with pain control, it facilitated activities that increased the quality of life. Where quality of life improved, reduced stressors and experiences of migraine-related guilt and shame were thought to help prevent the onset of agitation-related migraines. A notable exception here was Participant 6, who reported no benefits from the intervention. In discussing this outcome with the interviewer, Participant 6 attributed the lack of therapeutic benefit to the “random” nature of their migraines, noting that daily hassles, psychological overwhelm, and embodied agitation were not related to the onset, severity, or duration of their migraines. They were convinced that mindfulness-based interventions would only be effective for stress-based migraines and described entering into the study without any expectation of positive benefit. We return to the significance of this case in the discussion. In what follows, we consider how participants understood the role of Muse technology in these positive outcomes.

### Perceived Benefits and Drawbacks of Incorporating Muse Technology in Mindfulness Meditation in the Context of Migraine Treatment

Participants reported the benefits and drawbacks of incorporating live EEG feedback via Muse into their mindfulness meditation sessions. Participants largely spoke to the benefits of 2 Muse features. First, all except Participant 6 found the session-tracking feature beneficial for motivating adherence. Participant 1 noted, “the thing I really liked about [Muse^TM^] was that ... you got an objective recording that you did the thing.” Participants found seeing the record of completed exercises within the Muse app to be rewarding, sort of like a “a place to put the check mark” or an “objective recording that you did the thing” (Participant 1). Second, all participants found the live EEG data helpful for developing their meditation practice and tracking progress over time, such as how often and how long they meditated. For example, Participant 3 explained how the data on their mental activity provided objective documentation of a practice that can be difficult to monitor:

I think it’s easier to deny ... or pretend that I’m not in a moment of frenetic [mental] activity or thinking [without neurofeedback]. I can just tell my mind to be quiet and then think that I’m quiet whereas the Muse^TM^ neurofeedback—it’s tracking something very different, not what I’m saying to myself, but my actual brain waves ... it provided a different perspective on my being while I was meditating. I thought that was really helpful.

The EEG data offered valuable insights into their capacity to attain a meditative state, insights that could not necessarily be obtained through interoception alone. Participant 4 echoed this sentiment, finding the live EEG data helpful for making meditation practice more tangible and in so doing, easier and more enjoyable to learn: “[The Muse^TM^ EEG data] helped improve the experience because you could understand what happened at those certain times [when the mind was more active], and you could work to correct it, make it better, or do what you need to do to maybe limit that issue.” Without this neurofeedback, participants felt they would struggle to notice fluctuations in brain activity and track their progress over time.

Participants also reported negative aspects of meditation with the Muse device. These included technological and connectivity issues (eg, intermittent interruptions to the Bluetooth connection or inability to successfully pair the Muse device with the smartphone app), distraction and frustration related to EEG outputs, and headband discomfort. Technological and instrumental challenges were reported by all participants. Such issues were noted to be distracting and frustrating. Participant 5, who received a replacement band, explained, “when technology doesn’t work it does hinder your ability to fully focus and enjoy the process.” Connectivity issues not only detracted from the mindfulness meditation sessions but also threatened to dissuade consumer engagement altogether. As Participant 2 noted, it “takes time out of ... the meditation.”

Interestingly, while all participants reported that the summative EEG feedback was helpful for monitoring their progress across sessions, 8 participants found the live auditory neurofeedback alerts distracting, specifically, the bird chirping sounds indicating calmness and the storm sounds signaling heightened mental activity. These auditory cues, provided by the Muse device, were reported to be counterproductive to the users’ ability to maintain focus during meditation. They described negative feedback as particularly problematic, prompting frustration with one’s practice and further unsettling the mind. The emphasis on auditory rewards and punishments was also broadly criticized by 6 participants for contributing to the “gamification” of meditation in a way that negatively altered the practice experience. Participant 1 noted, “often the feedback was negative towards the experience ... when it was positive it was more so positive for the wrong reason ... almost like a gamification process and not so much, like, adding to the quality of the meditation.” For these reasons, 8 of the 10 participants described learning to tune out auditory feedback. Participant 2 explained:

I would try to almost chase the birds, which in turn caused more storms to come ... it was kind of like a negative feedback loop where I wanted the birds, but I was getting the storms, so I’d try hard to get the birds, but I’d get more storms ... [in time] I was able to ... let all that go ... if I heard birds, that was great. If I heard storms, cool.

Participant 3 similarly noted, “I know that part of the practice is not to judge where your mind is but just to accept and witness where it is ... sometimes I can accept where I’m at, sometimes I’m like, ‘No, I want to be calmer!’”

In total, 8 participants also reported that incongruencies between their perception of sessions and the information reflected in the EEG data bred feelings of frustration or disappointment. Participant 3 explained, “I was getting lots of storm noises, and not very many birds, and I was like, ‘what is going on here?’ ... the feedback was irritating in the sense that what I thought, or ... was expecting wasn’t actually happening.” Here again, participants reported learning to let go of negative emotions associated with this discrepancy and began to accept that their minds might be more active than they thought.

Finally, 5 participants found the Muse headband to be uncomfortable during active migraine symptoms. As Participant 4 noted, the added pressure of the band was a barrier to using the band during migraines: “that was difficult, because I know you need this, the data from the monitoring device, but when you have a migraine, that’s difficult to put something on your head. You don’t want to.”

Despite these critiques, all participants reported the ongoing use of mindfulness meditation techniques acquired during the intervention to manage their migraine disorders. Of the 10 participants, 7 expressed intentions to persist with mindfulness meditation using the Muse device. For these individuals, the benefits of neurofeedback outweighed any frustrations related to connectivity issues or other device limitations. They found the Muse device instrumental in assessing their progress over time. Moreover, each of these 7 participants expressed a desire to continue learning new meditation techniques and incorporate the mindfulness meditation practices acquired in the study into their daily routines.

## Discussion

### Principal Findings

In this study, we explored participants’ experiences of an 8-week neurofeedback-based mindfulness meditation intervention using the Muse device to manage migraine. The intervention aimed to understand whether neurofeedback-based mindfulness meditation could alleviate migraine symptoms and improve quality of life. Our findings indicate that the intervention was perceived as beneficial to most participants, leading to improvements in migraine experiences, including decreased migraine severity and frequency, improved coping and prevention skills, and overall improved quality of life. This included cognitive and emotional benefits, enhanced relationships, increased work performance, and the formation of healthier daily habits. At times, migraine and quality of life benefits were interconnected in complex, mutually supportive ways. For example, where reduced migraine severity led to fewer work absences, professional stress and guilt diminished. This net reduction in stress and other negative emotions was seen to help prevent intense migraine symptoms, compounding the benefits of the intervention.

In total, 9 of 10 participants also felt that neurofeedback-based mindfulness meditation with Muse improved their ability to manage migraine symptoms and prevent high-intensity attacks through (1) enhanced attentional control; (2) improved awareness of thoughts, bodily sensations, and migraine triggers; (3) developing the ability to engage in psychological decoupling (mechanism of disengaging the physical aspects of pain from the emotional aspects of pain, leading to a reduced experience of distress through nonattachment [4]); and (4) an enhanced ability to achieve physical and mental relaxation. Such changes point to perceptions of reduced migraine-related disability as a result of the neurofeedback-based mindfulness meditation intervention.

While our study was the first to use at-home neurofeedback-based mindfulness meditation training, our findings support previous research regarding the use of mindfulness meditation to treat migraine and other chronic pain conditions, revealing that alternative treatments for migraine can be beneficial for migraine pain and overall quality of life. Previous studies on mindfulness meditation note similar improvements to overall well-being as the current research, including improved management of pain [24], better sleep [52], enhanced cognitive function, including attention and motivation [53], and improved emotional well-being, lowering stress and tension [22]. The processes by which mindfulness meditation was believed to improve migraine experiences were also aligned with past research, with participants noting the importance of learning to disengage and view pain differently [54], increasing body awareness [5], enhancing personal agency [24], and reducing stress [55].

The study highlights the importance of fostering enhanced self-efficacy for individuals with migraine. For those living with chronic, unpredictable, and debilitating health conditions, the sense of one’s body being “out of control” often creates immense stress and tribulation [56-58]. As participants in our study noted, the sense of hopelessness and helplessness that can result from feeling powerless to predict, prevent, or manage physical pain and incapacity diminishes the quality of life and contributes to pain-related fear and migraine dread. Where those living with chronic illness can develop some sense of self-efficacy in understanding, predicting, or influencing extraordinary bodies, they foster a more constructive and optimistic relationship to migraines and a more empowered sense of self [57]. As past researchers have noted, mindfulness meditation interventions allow individuals with migraine to recognize warning signs of migraine and make decisions in their own treatment [5,6].

In addition to concrete shifts in pain severity, symptom frequency, mood, cognitive performance, and disability, participants also spoke to a more qualitative shift in the meaning and experience of migraine resulting from the intervention. By reframing migraine pain from a meaningless source of distress to a sign of overwhelm and the need for self-care and an indication that the person might need to make changes to everyday life, the illness experience is imbued with meaning and saved from radical antagonism and senselessness. Participants were able to construe painful bodies as attentive, caring bodies that bear witness to challenging life circumstances and provide clues about personal well-being. What emerges in these stories is the figure of the “able (hu)man” (sic)—who takes initiative, recognizes their own power, and actualizes their ability to intervene and persevere in their own life [59]. Such processes of narrative reconstruction and identity work to symbolically rescue those living with chronic pain and illness from tragedy and meaningless distress, reinfusing the migraine experience with hope and significance [59-63].

Current findings suggest that the Muse device had an overall positive impact on participants’ experiences of the mindfulness meditation intervention. The primary benefits of this device were that it provided structure and accountability to the mindfulness meditation practices, keeping participants on track by providing daily data on the number of days they had meditated. Another benefit was the objective recording of their EEG data, showing the quality of their mindfulness meditation sessions. This was approached with positive attitudes, as it provided the reality rather than personal impressions of their sessions. This motivated participants to persist in the intervention, leading to participants striving for a less active mind during future mindfulness meditations. In addition, participants perceived drawbacks from the Muse technology, including connectivity issues, frustration with the feedback system, and concerns of the accuracy of the EEG data. There are improvements that could be made with this technology in order for participants to feel confident about using the Muse during their meditation sessions. However, the majority of participants listed the desire to continue mindfulness meditation with Muse in the future.

The study participants noted multiple benefits and drawbacks of the Muse technology, where the positive outcomes reported suggest that neurofeedback-based mindfulness meditation with Muse technology could be a beneficial treatment tool for some individuals with migraine. The 1 divergent experience (Participant 6, who reported no benefits from the intervention and attributed their lack of success to the fact that their migraines were not stress-related) suggests the importance of understanding the causal ontologies affirmed by specific individuals with migraine to support the identification of best candidates. For example, those who do not consider particular triggers (such as stress) to be relevant to their migraine experiences are less likely to adhere to behavioral treatment modalities that target such triggers [64,65]. At the same time, it is well-established that beliefs about treatment effectiveness affect treatment outcomes [66]. Given these findings, greater attention to how endorsement of the “stress” hypothesis and beliefs about intervention effectiveness impact neurofeedback-based mindfulness meditation use, adherence, and outcomes among individuals with migraine is warranted.

The burden of living with migraine often results in individuals with migraine receiving pharmaceutical treatments, which are limited in a number of ways. Pharmaceutical treatments can lead to a reliance on medications and medication-overuse headaches [2]. In addition, pharmaceutical treatments should not be used by individuals living with stomach problems and who are pregnant or breastfeeding [2]. These limitations exhibit why alternative treatments, such as neurofeedback-based mindfulness meditations, should be available for individuals with migraine. Our study supports the growing body of research demonstrating the benefits of mindfulness meditation, such as improved management of chronic pain, emotional well-being, cognitive functioning, and overall perception [20,22,53,67,68]. These improvements have been found in quantitative research on alternative treatments of migraine, resulting in decreased migraine pain and disability and helping individuals recognize early warning signs of migraine [4,69]. This study expands on these findings from a qualitative perspective, revealing the importance of the lived experiences of individuals with migraine. This study reveals the importance of agency for individuals with migraine, as having a sense of control over their chronic disease allowed them to separate themselves from their migraine.

### Limitations and Future Research

Several methodological limitations are worth noting. First, the original efficacy study excluded participants who regularly used preventative medication and who already practiced meditation (prior to the study). It is unclear if participants used other evidence-based migraine treatments (eg, botulinum toxin), and if so, to what extent. Second, a migraine diary was used to collect data on migraine frequency and symptoms. There is some evidence that a migraine diary alone can improve migraine symptoms and coping by making individuals with migraine more aware of their triggers [60]. Thus, reported improvements in migraine within this study cannot be attributed solely to the combined neurofeedback and mindfulness meditation intervention. Third, our sample size was limited and notably lacking in ethnic diversity. Finally, participants were interviewed at different times, ranging from a month to over a year after the original efficacy study. Results may be impacted by memory bias or the propensity to recall memories that are more congruent with current emotional states [70]. Interim factors like continued or discontinued use of the intervention, changes in migraine condition, and external influences (eg, lifestyle changes and medication use) may have also biased participants’ perceptions of the intervention.

To our knowledge, this is the first study to examine a self-administered neurofeedback and mindfulness meditation intervention for migraine. Future research is needed to validate the effectiveness of this treatment protocol, control for potential confounds, and optimize the intervention. Follow-up studies with larger and more diverse participant groups would be beneficial for commenting on the generalizability of our findings and investigating how individual differences (symptoms, daily activities, and personal characteristics) might impact experiences of neurofeedback-based mindfulness meditation interventions for individuals with migraines. Longitudinal studies are needed to explore the effects of this intervention over time. Finally, the limitations of the Muse system noted by participants highlight the value of considering user input and perspectives. Future studies should explore how different user settings might benefit experiences and adherence rates and how to best reduce technological difficulties and related user frustrations. For example, future studies might consider whether the ability to omit (mute) distracting auditory cues enhances user experiences or increases intervention adherence. Biofeedback output could also be improved to make it more suitable for individuals with sensory disorders (eg, vibration instead of auditory cues). Moreover, the Muse system could address technical issues that cause difficulties in calibrating the device for participants and enhance the electrodes to ensure that they function more effectively. Continued exploration of user expectations and experiences is crucial to better understand the acceptability and feasibility of these mobile health tools and design quality consumer-grade migraine products that meet the needs and health objectives of diverse migraine experiences.

### Conclusions

Notably, as the first study to evaluate the experiences of individuals with migraine in an at-home, neurofeedback-based mindfulness meditation intervention, this investigation adds to our understanding of nonpharmaceutical migraine treatment. The skills learned by participants in this intervention led to the perception of improved migraine symptoms and improvements in quality of life. This perception was brought on by an increased sense of agency, leading to a narrative shift toward a body that provides useful early warning signs and valuable insights into daily life and a self that is able to interpret and influence embodied distress. While changes in the severity and frequency of migraine pain as a result of the intervention are evident to greater or lesser degrees, this increased sense of agency also led to a higher sense of control over one’s migraine, which can result in positive outcomes for coping with this disease. As the individual was transformed from a state of helplessness and hopelessness to one of increased agency and optimism, these experiences led to a profound shift in their global experience of migraine disorder. In addition, this type of treatment for migraine is safe and effective with few to no negative side effects, as the Muse device and mindfulness meditation have minimal negative implications. Consequently, this treatment should be considered on its own for individuals who are unable to take pharmaceutical treatments for migraine. Additionally, this treatment should be considered alongside medication in order to learn the valuable coping and prevention techniques acquired in this neurofeedback-based mindfulness meditation intervention. Ideally, this would lead to individuals with migraine putting more reliance on mindfulness meditation for the treatment of migraine and reduce their medication intake. Ultimately, this study revealed that neurofeedback-based mindfulness meditation can be a valuable intervention for migraine, providing possibilities beyond pharmaceutical treatments.

## Abbreviations

AA: anticipatory anxiety
EEG: electroencephalogram
